# An Integrative View of Mechanisms Underlying Generalized Spike-and-Wave Epileptic Seizures and Its Implication on Optimal Therapeutic Treatments

**DOI:** 10.1371/journal.pone.0022440

**Published:** 2011-07-21

**Authors:** Boyuan Yan, Peng Li

**Affiliations:** 1 Department of Electrical and Computer Engineering, Texas A&M University, College Station, Texas, United States of America; 2 Department of Electrical and Computer Engineering, Texas A&M University, College Station, Texas, United States of America; Mount Sinai School of Medicine, United States of America

## Abstract

Many types of epileptic seizures are characterized by generalized spike-and-wave discharges. In the past, notable effort has been devoted to understanding seizure dynamics and various hypotheses have been proposed to explain the underlying mechanisms. In this paper, by taking an integrative view of the underlying mechanisms, we demonstrate that epileptic seizures can be generated by many different combinations of synaptic strengths and intrinsic membrane properties. This integrative view has important medical implications: the specific state of a patient characterized by a set of biophysical characteristics ultimately determines the optimal therapeutic treatment. Through the same view, we further demonstrate the potentiation effect of rational polypharmacy in the treatment of epilepsy and provide a new angle to resolve the debate on polypharmacy. Our results underscore the need for personalized medicine and demonstrate that computer modeling and simulation may play an important role in assisting the clinicians in selecting the optimal treatment on an individual basis.

## Introduction

Generalized spike-and-wave (SW) patterns of the electroencephalogram (EEG) are typically observed during many types of epileptic seizures, in particular, during absence seizures, which are characterized by clear-cut spike-and-wave EEG oscillations. The mechanisms underlying spike-and-wave patterns are complex and may involve cerebral cortex and thalamus, intrinsic properties of neurons, and various types of synaptic receptors present in the circuit. There has been notable effort devoted to understanding seizure dynamics and various hypotheses have been proposed to explain the underlying mechanisms [1], [2]. Some studies [3]–[7] demonstrate that synaptic receptors are especially important in the generation of epileptic seizures while others believe intrinsic properties of neurons play an important role [8]–[14]. While each of such hypotheses is supported by some experimental evidence, they tend to only cover a small subset of underlying causes and may even appear contradictory to each other.

It is well known in the field of neuroscience that virtually indistinguishable network activities may arise from widely disparate sets of underlying mechanisms [15]–[17]. As far as spike-and-wave oscillations are concerned, it is not difficult to foresee the existence of a complex parameter landscape. In this paper, a thalamocortical model is developed based on [5], [7] to encompass key interplays between the thalamus and cortex as well as within the cortex through inhibitory, excitatory synaptic receptors and intrinsic currents.

The first half of the paper is devoted to understanding the interplay of various mechanisms underlying generalized spike-and-wave epileptic seizures by taking an integrative view. To achieve this goal, we first study the interplay of inhibitory and excitatory receptors on specific synaptic connections and our results demonstrate that: (1) although both GABA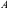 and GABA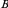 receptors are actively involved in seizure generation, they play quite different roles: while the former typically inhibits seizure activity, the latter actually induces the absence-like seizure activity; (2) while intuition may suggest that increased excitation would cause seizures, the interplay of AMPA-type glutamate receptors can be complex and seizures may even be generated by decreased excitation; (3) intrinsic properties of neurons also have a significant impact on seizure generation. In addition to the roles of specific synaptic connections, the global role played by a given type of synaptic receptor in the whole network is also studied. We demonstrate how network behavior is influenced by changes in the efficacy of all the synapses mediated by the same type of receptor. The results have direct implications on the study of drug treatment of epilepsy. Finally, we performed one million simulations to show the interplay of multiple mechanisms in a high dimensional parameter space. For each simulation, all the synaptic conductances were randomly generated following a unform distribution within specified ranges. The distribution of the simulation results demonstrates that both spindle and spike-and-wave activities can be produced by a huge number of parameter combinations and both activities cover the full range of tested values of all the synapses.

In the second half of the paper, we study the implications of the integrative view of mechanisms underlying epileptic seizures on the optimal epilepsy treatments. For a given activity of the network, the underlying parameter combinations form a complex parameter set in the high-dimensional parameter space, where each point in the parameter set of spike-and-wave oscillations can be considered as a specific *pathological instance*, which corresponds to a particular individual suffering from epilepsy. There has been widespread debate about the effect of polypharmacy for the treatment of epilepsy [18]. While some researchers think that the combination of two low-dose drugs generates a greater therapeutic effect with less risk of toxicity than a larger dose of a single drug [19]–[23], others believe that a full dose of one drug achieves better control of seizures with fewer adverse effects [24]–[28]. In this paper, we provide a more complete picture and show that the location of each pathological instance in the parameter space is an important factor to consider when determining the best treatment with the minimal drug dose. To provide a quantitative assessment of the optimal therapeutical treatment, we formulate and solve a mathematical optimization problem.

Our results in the second part of the paper show that: (1) the optimal treatment is a function of the state of individual patients as well as the potency of the candidate drugs; (2) the potentiation of rational polypharmacy for drugs with complementary receptors can be explained by the non-linearity of the drug-response characteristics; (3) while monopharmacy is optimal for the majority of patients, rational polypharmacy is required by a small portion of patients represented by pathological instance points located deeply inside the pathological regime in the parameter space. (4) our study foresees the benefits of personalized medicine, a practice in which computer modeling and simulation assist the clinicians in determining the optimal treatment on an individual basis.

## Results and Discussion

**Figure 1:**
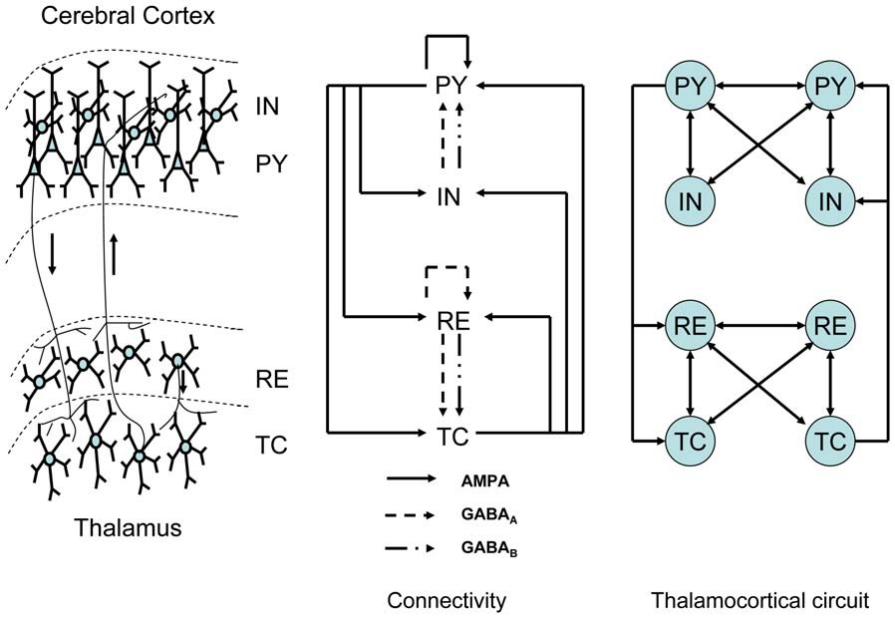
Thalamocortical network model.

An illustration of the thalamocortical network, its synaptic connectivity and the basic network model with eight cells are shown in Fig. 1. The thalamus is modeled by the thalamic reticular (RE) and thalamocortical (TC) cells, and the deep layers of the cortex are represented by the pyramidal (PY) and interneurons (IN) cells. This simple eight cell model is proven to be able to make predictions for larger networks with similar structures [7] (see details in Materials and Methods).

**Figure 2:**
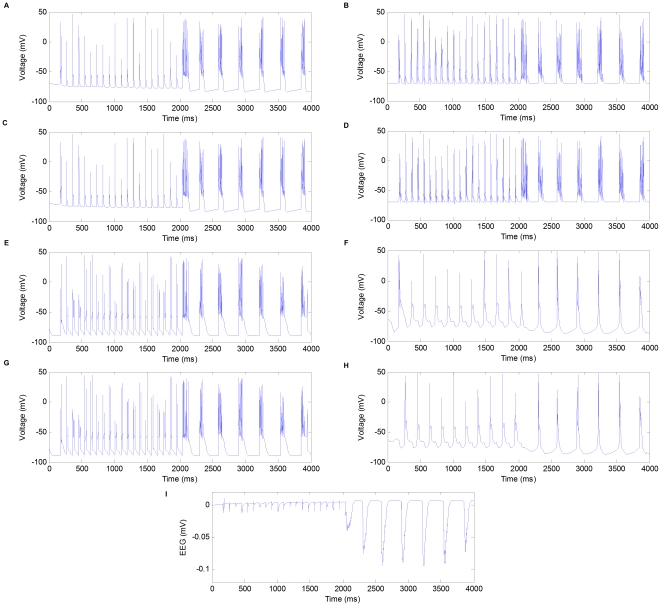
The firing activities of different types of neurons and field potentials in the transition from spindles to spike-and-wave by blocking synaptic GABA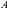 receptors in cerebral cortex at 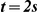. (A) Firing activities of the first pyramidal cell (PY). (B) Firing activities of the first interneuron (IN). (C) Firing activities of the second pyramidal cell (PY). (D) Firing activities of the second interneuron (IN). (E) Firing activities of the first reticular cell (RE). (F) Firing activities of the first thalamocortical cell (TC). (G) Firing activities of the second reticular cell (RE). (H) Firing activities of the second thalamocortical cell (TC). (I) Field potentials.

Fig. 2 shows the firing activities of different types of neurons and field potentials of the network in the transition from spindles to spike-and-wave by blocking synaptic GABA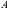 receptors in cerebral cortex at 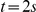.

In the first two seconds of the simulation, the network is in the control condition and generates the typical synchronized spindle oscillations at about 10 Hz. Exemplary firing patterns of the cells are shown in Fig. 2, where the TC cells discharge once every two cycles and all other cells discharge in every cycle. These simulated activities are consistent with typical spindle oscillations observed experimentally [29], [30]. The corresponding field potentials are shown in Fig. 2I (in the first two seconds of the simulation), which are characterized by successive negative deflections at a frequency of about 10 Hz.

Starting from 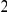 seconds, as the synaptic GABA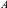 receptors in cerebral cortex are blocked(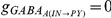), the network transits from the spindle oscillations to a slower oscillation mode (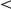
4 Hz) with field potentials characterized by large-amplitude negative spikes and small-amplitude positive waves. The spike-and-wave(SW) patterns are typically observed during epileptic seizures. In the last two seconds of simulation in Fig. 2, all the cells fire prolonged high-frequency discharges synchronously during the negative spikes and the positive waves are coincident with the silent periods of all the cells. This portrait is typical of experimental recordings of cortical and thalamic cells with the SW oscillation pattern.

All the values of parameters in the control condition are provided in Materials and Methods. In the following sections, the default value of the control condition is chosen for each parameter unless stated otherwise.

### An integrative view of mechanisms underlying generalized spike-and-wave epileptic seizures

In the previous demonstration, the transition from spindles to SW is obtained by blocking GABA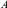 receptors in the cortex. However, the transition can also be triggered by alterating other parameters. Indeed, the activities of the network (spindles, SW, etc.) are determined by the combination of a large number of parameters including both synaptic receptors and intrinsic currents of neurons. In this section, we take an integrative view of various mechanisms underlying generalized spike-and-wave epileptic seizures and demonstrate that the epileptic activities are resulted from the interplay of a large number of underlying parameters.

Before proceeding further, we first classify the typical behaviors of the network into one of the five categories:

1. **RT** (resting mode): resting state.
2. **SP** (spindle): 7–15 Hz oscillation; PY cells generate a few spikes per cycle, and the field potential consists of successive positive and negative deflections.
3. **DE** (delta): 1–4 Hz oscillation; PY cells generate a few spikes per cycle, and the field potential consists of successive positive and negative deflections similar to that of spindles but at lower frequencies.
4. **SW** (spike-and-wave): 1–4 Hz oscillation; PY cells generate prolonged firing per cycle, and the field potential consists of large-amplitude negative spikes and small-amplitude positive waves.
5. **EF** (extreme prolonged firing): an extreme case of prolonged firing; due to overwhelming excitation, such prolonged firing will continue forever.

**Figure 3:**
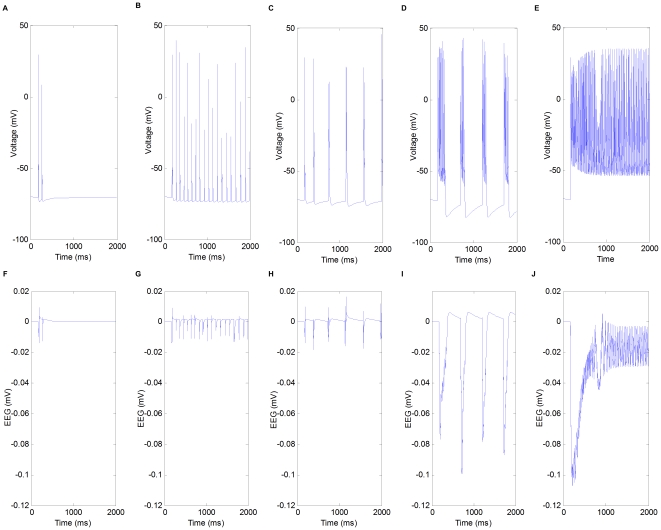
Firing activities and field potentials in different modes of the network. (A) Firing activities of pyramidal cells in resting mode (RT). (B) Firing activities of pyramidal cells in the mode of spindle oscillation (SP). (C) Firing activities of pyramidal cells in the mode of delta oscillation (DE). (D) Firing activities of pyramidal cells in the mode of spike-and-wave (SW). (E) Firing activities of pyramidal cells in the mode of extreme prolonged firing (EF). (F) Field potentials in resting mode (RT). (G) Field potentials in the mode of spindle oscillation (SP). (H) Field potentials in the mode of delta oscillation (DE). (I) Field potentials in the mode of spike-and-wave Mode (SW). (J) Field potentials in the mode of extreme prolonged firing (EF).

The firing activities and field potentials in different modes of the network are shown in Fig. 3. Generally speaking, as the balance between excitation and inhibition shifts towards excitation, the modes of the network tend to shift from (1) to (5) accordingly. Note that, the resting state of the brain is only a “theoretical mode” in the study, which does not really exist. In reality, neurons are always active one way or the other due to external sensory inputs, more intrinsic membrane properties, and many other possible mechanisms.

In the following sections, RT, SP, DE, SW, and EF will be used to denote the corresponding behaviors as defined above.

#### Interplay of GABA-mediated inhibitions

Gamma-aminobutyric acid(GABA) is the chief inhibitory neurotransmitter in the central nervous systems vertebrates. There are two classes of GABA receptors: GABA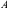 receptor (ligand-gated ion channels), which responds fast to GABA, and GABA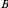 receptor (G protein-coupled receptors), which responds slowly to GABA. Although both are important in seizure generation, they are playing quite different roles. While GABA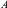 receptor is generally believed to inhibit seizure activity, GABA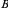 receptor has been shown to induce absence seizure like activity [31]–[34]. As a result, while many anti-absence drug (clobazam, clonazepam, pheobarbital, primidone, etc.) are designed as GABA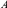 agonists to inhibit seizures, GABA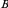 antagonists hold the promise as anticonvulsants for absence seizures.

The role of GABA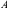-mediated inhibition, GABA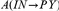, was initially demonstrated in Fig. 2. In this section, we extend our studies to two more important GABA-mediated inhibitions in the thalamus: intra-RE GABA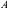-mediated inhibition GABA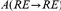 and intrathalamic GABA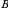-mediated inhibition GABA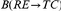. While some key properties were studied in [5], we provide a more complete picture by examining the interplay between the two inhibitions and GABA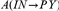.

#### Intra-RE GABA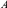-mediated inhibition: GABA
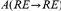


**Figure 4:**
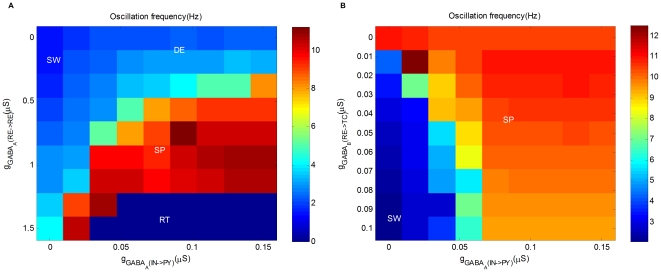
The interplay of GABA-mediated inhibitions in the generation of epileptic seizures. (A) Oscillation frequency as a function of maximal synaptic conductance 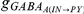 (X-axis) and maximal synaptic conductance 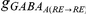 (Y-axis). (B) Oscillation frequency as a function of maximal synaptic conductance 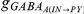 (X-axis) and maximal synaptic conductance 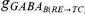 (Y-axis).

As shown in Fig. 4A, the parameter space of 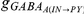 and 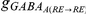 is divided into four regions characterized by different modes of network behavior. When 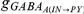 is small, the network is in the mode of SW. When 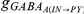 is large, the network successively displays the modes of DE, SP, and RT as 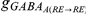 increases. The transition from SP to DE made by blocking GABA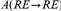 was demonstrated in the study of [5], which is in agreement with *in vivo* injections of bicuculline into the thalamus [30]. This suggests that, given a large 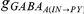, the suppression of GABA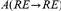 may only affect the oscillation frequency but does not generate SW because the pyramidal cells are still under the control of GABA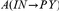.

With the more complete picture in Fig. 4A, it is evident that while both DE and SP can be transited into SW by decreasing GABA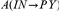, it is easier to generate epileptic seizures starting from DE. Moreover, for fixed GABA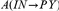, epileptic seizures are easier to occur with less GABA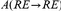. As a result, reinforcing GABA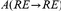 could reduce the tendency to generate epileptic seizures, which is in agreement with the presumed role of anti-absence drug clonazepam [35]. Note that, while a moderate increase of GABA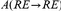 can resist the occurrence of epileptic seizures, Fig. 4A also indicates that too much GABA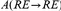 may drive the network into RT and thus affect the normal function of the brain.

#### Intrathalamic GABA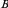-mediated inhibition: GABA
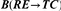


In addition to GABA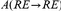, GABA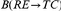 is also believed to play an important role in the generation of epileptic seizures due to the nonlinear response property of GABA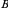 type receptors [7], [33]. As shown in Fig. 4B, the parameter space of 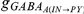 and 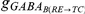 includes two regions: SW and SP. It is clear that reducing 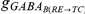 significantly diminishes SW in favor of SP, and increasing the conductance has the opposite effect. Especially, when 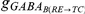 is 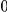, the thalamus can not be recruited into the mode of slower oscillation even if GABA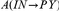 is suppressed to zero. This agrees with the fact that GABA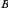 antagonists have been found useful in preventing the development of SW in some animal models [33].

Studies based on the current model suggest that the generation of epileptic seizures needs the involvement of both cortex and thalamus [7]. Thalamus plays an important role as it can oscillate at low frequencies (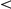
4 Hz) due to the post-inhibitory rebound bursting property of TC cells. TC cells are capable of firing a burst of action potentials in response to inhibitions from RE cells, mediated by both GABA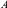 and GABA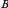. The slowly activated GABA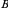 receptors make it possible for thalamus to perform oscillations at low frequencies (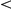
4 Hz). As a result, with GABA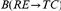 available, an epileptic seizure can be triggered by suppressing GABA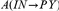. Otherwise, as demonstrated in Fig. 4B, the seizure will not happen even if GABA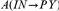 is suppressed to zero.

In addition, recent studies have also suggested the neocortical origin of spike-and-wave field potentials in experimental models of absence seizure [36], [37]. Without thalamic participation, intrinsic rebound mechanisms of cortical cells as those in TC cells may be necessary in order to generate sustained spike-and-wave oscillations. One such candidate is the low-threshold spike (LTS) pyramidal cells in cerebral cortex, which could respond to hyperpolarization with a burst of action potentials. With LTS PY cells, there is a loop inside the cortex: PY cells excite IN cells, IN cells inhibit LTS PY cells, and LTS PY cells produce rebound burst firings to start the next cycle of the sustained oscillation. Computational studies [38] have demonstrated that spike-and-wave oscillations can be generated in a network of cortical neurons, where 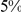 of PY cells are LTS PY cells. In the current work, we study the role of thalamocortical loops in epileptic seizure generation and the LTS PY cells are not introduced in our current model.

#### Interplay of AMPA-mediated excitations

While the earlier studies of [5], [7] focused on inhibitory receptors, AMPA-type glutamate receptors, which mediate fast excitatory neurotransmission, also play an important role in the generation of epileptic seizures. Several antiepileptic drugs are designed as AMPA antagonists like talampanel and perampanel [39], [40]. While intuition may suggest that increased excitation would cause seizures, the interplay of AMPA-mediated excitations could be complex and seizures could even be generated by decreased excitations. This implies that special caution should be taken in the drug design process. To illustrate this point, the interplay of two intracortical AMPA-mediated excitations, AMPA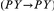 and AMPA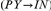 are studied in this section.

#### Intra-PY AMPA-mediated excitation: AMPA
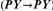


**Figure 5:**
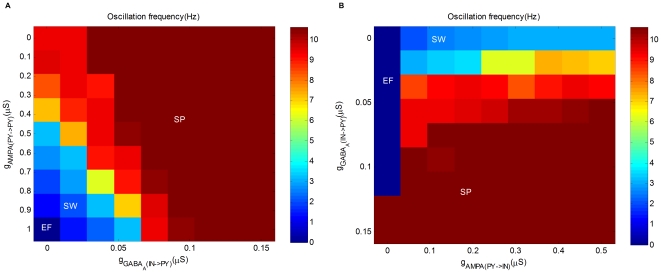
The effect of AMPA-mediated excitations in the generation of epileptic seizures. (A) Oscillation frequency as a function of maximal synaptic conductance 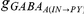 (X-axis) and maximal synaptic conductance 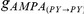 (Y-axis). (B) Oscillation frequency as a function of maximal synaptic conductance 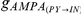 (X-axis) and maximal synaptic conductance 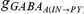 (Y-axis).

While suppressing 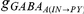 has been shown to be an effective way to generate seizure, Fig. 5A indicates an alternative path to seizure by increasing excitation AMPA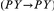. The modes of the network are determined by the balance between excitation and inhibition characterized by the relative strengths of 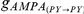 and 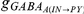. Generally speaking, reducing 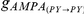 or increasing 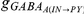 can significantly diminish SW in favor of SP, and increasing 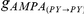 or reducing 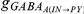 has the opposite effect. This also indicates that anti-absence drugs may be designed as AMPA antagonists. Note that, as shown in the upper part of Fig. 5A, when 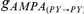 is small, the network can not be transited into SW even if GABA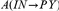 is suppressed to zero.

#### PY-to-IN AMPA-mediated excitation: AMPA
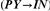


Although both are excitatory, different from AMPA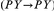, AMPA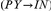 plays an opposite role. Intracortically, the firing of PY cells results in excitatory postsynaptic potentials on both PY and IN cells due to AMPA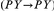 and AMPA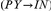, respectively. While the former contributes to the excitation of PY cells directly, the latter enhances the activity of IN cells, which produce inhibitory postsynaptic potentials on PY cells. Therefore, AMPA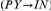 contributes to the inhibition of PY cells indirectly. As shown in Fig. 5B, increasing 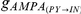 diminishes SW in favor of SP.

**Figure 6:**
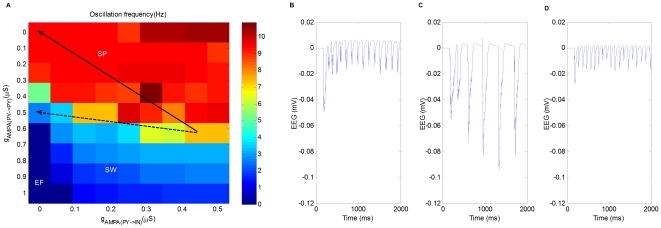
The suppression of both synaptic excitations in cortex in the generation of seizures. (A) Oscillation frequency as a function of maximal synaptic conductance 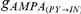 (X-axis) and maximal synaptic conductance 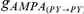 (Y-axis). If 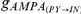 and 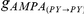 are decreased “proportionally”, the network will remain in SP (solid arrow). However, if the decreases of the conductances are out of proportion, it is possible for the network to transit from SP to SW (dashed arrow). (B) The field potential of the starting point of both arrows (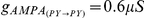, 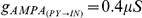). (C) The field potential of the ending point of the dashed arrow (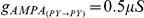, 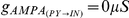). (D) The field potential of the ending point of the solid arrow (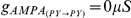, 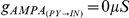). The results in Fig. 6 are obtained with 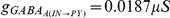.

While it is generally true that the suppression of 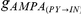 may lead to epileptic seizures, the suppression of both AMPA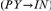 and AMPA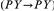 can also lead to epileptic seizures. The parameter space of 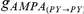 and 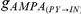 is shown in Fig. 6A. If 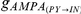 and 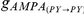 are decreased “proportionally”, the network will remain in SP (solid arrow). However, if the decreases of the two conductances are out of proportion, it is possible for the network to transit from SP to SW (dashed arrow). The field potentials of the starting point and the ending points of both arrows are shown in Fig. 6B, Fig. 6C, and Fig. 6D, respectively.

This complex interplay between AMPA-mediated excitations demonstrates another path to seizure, which is different from the common notion that the seizure is caused by an increase of the strength of excitatory synapses. Similar results were reported in computational studies [41], [42] and supported by experimental observations [43]. These results suggest that special caution should be taken in the drug design process.

#### Cellular mechanisms of intrinsic currents

Intrinsic membrane currents affect the network-level oscillatory behavior in an important way. In the study of [5], [7], a small set of ion channels are used to characterize pyramidal cells which generate the regular spiking pattern. Nevertheless, there exists a wide variety of ion channels and many of them are believed to play important roles in the generation of seizures [8]–[14]. Especially, some of the ion channels are important targets for anti-epileptic drug design. Towards a more complete understanding of the effect of intrinsic membrane currents, we introduce several key ion channels to the model and demonstrate the interplay between synapses and intrinsic membrane properties of neurons.

#### Persistent 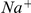 current: 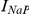


**Figure 7:**
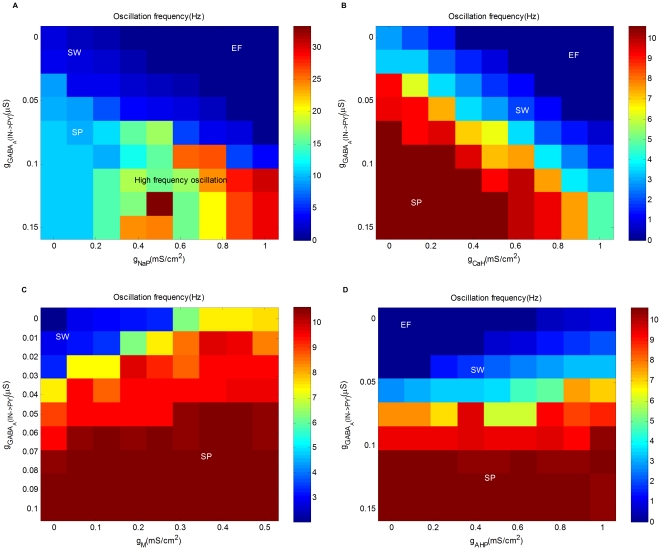
Cellular mechanisms of intrinsic currents. (A) Oscillation frequency as a function of the maximum conductance of persistent sodium 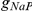 (X-axis) and the maximal synaptic conductance 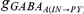 (Y-axis). (B) Oscillation frequency as a function of the maximal conductance of high threshold calcium 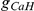 (X-axis) and the maximal synaptic conductance 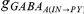 (Y-axis). (C) Oscillation frequency as a function of the maximum conductance of M-current 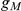 (X-axis) and the maximal synaptic conductance 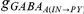 (Y-axis). (D) Oscillation frequency as a function of the maximal conductance of afterhyperpolarization current 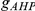 (X-axis) and the maximal synaptic conductance 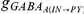 (Y-axis). The results in Fig. 7D are obtained with 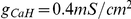.

One important intrinsic current to consider is the persistent 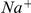 current 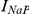. 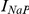 is a small slowly-inactivating 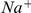 current with kinetics of inactivation in tens of seconds. This current may amplify synaptic potentials, generate subthreshold oscillations, facilitate repetitive firing, and maintain prolonged depolarized potentials. As epilepsy is associated with 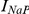 of an amplitude several times larger than typically observed under the normal physiological conditions, 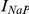 is believed to contribute to the pathophysiological hyperexcitability associated with the disorder. As shown in Fig. 7A, the parameter space of 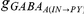 and 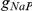 is divided into four regions: SW, SP, EF, and high frequency oscillation (which oscillates at higher frequencies than spindles due to the existence of NaP). As 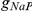 increases, the region of SW expands significantly. This implies that epileptic seizures may happen even with strong inhibition if the excitation is enhanced by the activation of 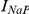. As a result, the persistent sodium current is a common target of anti-epileptic drugs, and can be reduced by a wide variety of anti-epileptic drugs [44]–[48].

#### High-threshold calcium current: 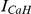


Another intrinsic current that plays a similar role is the high-threshold calcium current 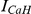. Similar to 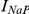, 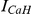 is also slowly inactivating. The key difference between the two is that 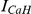 is activated only at a higher threshold voltage and thus is often triggered by action potentials. After activation, it contributes significantly to the depolarization of the membrane, thus amplifies synaptic potentials and maintains prolonged depolarized potentials. The parameter space of 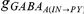 and 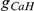 is depicted in Fig 7B, where the region of SW expands significantly as 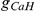 increases. Some anti-epileptic drugs have been proposed to antagonize high-threshold calcium channels including phenytoin [49], carbamazepine [50], topiramate [51], etc.

#### M-current: 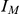


So far, we have demonstrated the effects of two intrinsic currents, which serve as enhancers of neuronal excitability. On the other hand, some other intrinsic currents may play different roles in determining the mode of network oscillation. One such intrinsic current is the M-current 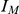, which is a slowly activating potassium current. The most important feature of 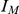 is that a significant amount of this current is on near the resting potential. As a result, 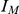 usually acts as a damper on neuronal excitability. As shown in the parameter space of 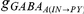 and 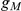 depicted in Fig 7C, 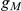 has the similar effect of 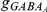: increasing 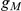 diminishes SW in favor of SP and decreasing the conductance has the opposite effect. As a result, M current can also be a target of anti-epileptic drugs. For example, retigabine (and ICA-27243) enhances M-current activation through the voltage-gated 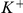 channel 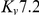 (also known as KCNQ2) [39], [52].

#### Afterhyperpolarization current: 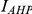


Another important intrinsic current is the afterhyperpolarization current 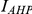, which is a potassium current activated by the calcium current during an action potential. Due to the non-inactivating property and large time constant, the amount of 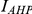 activated by bursts of action potentials is easily accumulated and produces long-lasting hyperpolarizations after the burst. As a result, 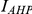 can play the role of the GABA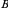 current 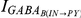 in generating long-lasting hyperpolarizations associated with the ‘waves’ component of SW. To demonstrate this, we add 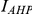 to the model and set 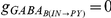 to disable the cortical GABA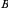 receptors. The parameter space of 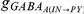 and 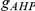 is depicted in Fig. 7D. When 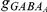 is large, the network is in the mode of SP. When 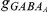 is small, the network has two different modes: SW and EF. As 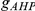 decreases, the balance between excitation and inhibition shifts towards the former. As a result, the range of SW decreases while the range of EF increases.

#### The interplay of mechanisms of different types

The interplays of a number of parameters have been studied pairwise in the previous sections. Those parameters can be divided into three groups: inhibitory synapses, excitatory synapses, and intrinsic ion channels, which have been widely found in many parts of the brain.

The roles of synaptic connections with specific presynaptic and postsynaptic neurons have been demonstrated. In addition to studying such specific synaptic connections, it is also important to study the global role played by a given type of synaptic receptor in the whole network. It is interesting to see how network behavior is influenced by changes in the efficacy of all the synapses mediated by the same type of receptor. It has direct implications on the study of drug treatment of epilepsy. For example, if an AMPA antagonist is applied, instead of a specific AMPA-mediated synapse, the strength of all the AMPA-mediated synapses in the network will be decreased.

In this section, we use AMPA and GABA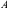 receptors together with persistent sodium ion channels as an example to study the global roles played by different synaptic receptors and ion channels in the whole network. In this study, the strength of all the AMPA-mediated synapses, GABA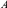-mediated synapses, and persistent sodium ion channels will be scaled by a factor of 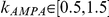, 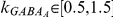, and 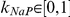, respectively. As a result, the scaled maximum conductances of all the AMPA receptor channels, GABA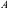 receptor channels, and persistent sodium ion channels will be in the ranges of 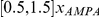, 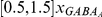, and 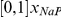, respectively, where 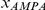 and 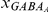 represent the corresponding values in the control condition and 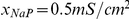.

**Figure 8:**
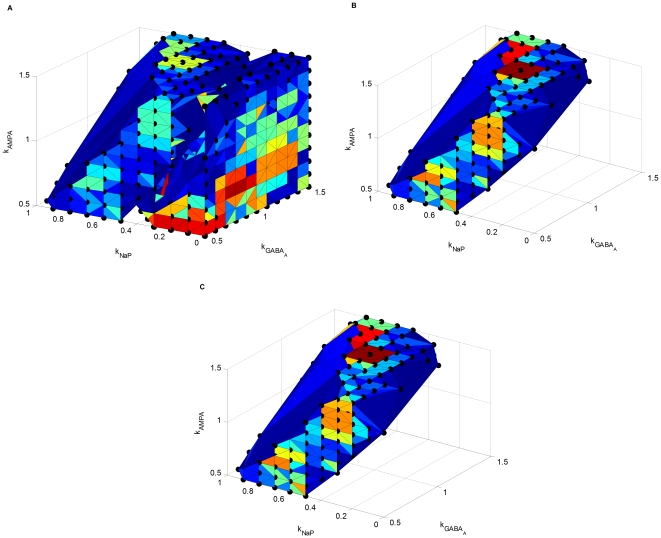
Pathological and physiological regimes in a three-dimensional parameter space. X-axis represents the scale factor of the maximum conductances of persistent sodium channels, 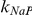, Y-axis represents the scale factor of the maximal conductances of GABA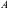 receptor channels, 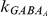, and Z-axis represents the scale factor of the maximal conductances of AMPA receptor channels, 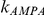. (A) Pathological and physiological regimes together. (B) Pathological regime. (C) Physiological regime.

The three scale factors are used as parameters and the corresponding parameter space is depicted in Fig. 8A, where the whole space is divided into two regimes corresponding to different modes of network oscillation: a pathological regime 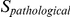 characterized by the spike-and-wave oscillation pattern as shown in Fig. 8B, and a physiological regime 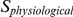 characterized by spindles as shown in Fig. 8C, respectively. The state of the network is determined by the interplay of three scale factors. Generally speaking, if the overall effect of excitation (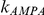 enhanced by 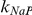) is more dominant than that of inhibition (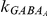), the state of the network is in the pathological regime. Otherwise, the state is in the physiological regime. Increasing 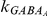, decreasing 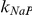, or decreasing 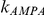 can diminish SW in favor of spindles.

#### The interplay of multiple mechanisms in a high dimensional parameter space

So far, we have only considered analysis with two or three parameters. In this section, we show the interplay of all the synaptic receptors in the network in a twelve-dimensional parameter space. The maximum conductances of the twelve synaptic receptor channels in the network are varied in the range of 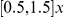, where 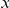 represents the corresponding maximum conductances in the control condition.

**Figure 9:**
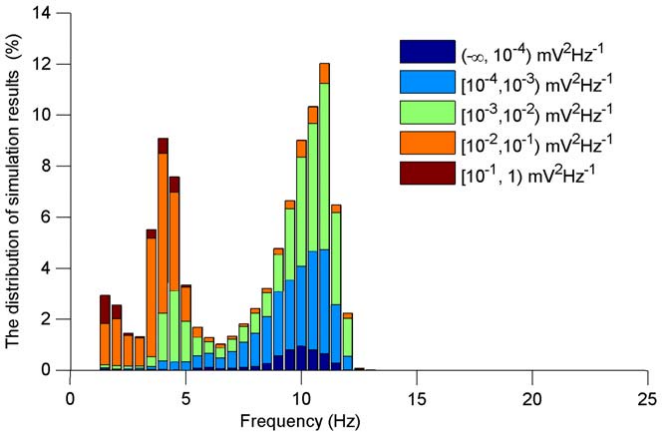
The distribution of the simulation results in terms of oscillation frequencies and magnitudes. X-axis represents the dominant frequency, Y-axis represents the percentage of simulations, and different colors represent different ranges of the magnitudes of the dominant frequency component as specified in the legend.

One million simulations are run and for each simulation all the parameters are randomly generated following a unform distribution within the ranges specified. Spectrum analysis is used after each simulation to obtain the most dominant harmonic component of the time-domain waveform of the field potentials. Fig. 9 shows the distribution of the simulation results in terms of the dominant frequencies. As shown in Fig. 9, most simulation results fall into the ranges of either 7–15 Hz oscillation or 1–4 Hz oscillation. In addition, while the former is mostly represented by blue or green colors, the latter is mostly represented by red or yellow colors. This means that the magnitudes of most 1–4 Hz oscillations are one or two orders larger than the magnitudes of 7–15 Hz oscillations.

Generally speaking, the 7–15 Hz oscillations with large magnitudes (green) represent spindle oscillations (SP), the 7–15 Hz oscillations with small magnitudes (blue) represent the resting mode (RT) (after few cycles of spindle oscillation at the initial stage, the network returns to resting state due to strong inhibition), the 1–4 Hz oscillations with small magnitudes (green) represent delta oscillations (DE), and the 1–4 Hz oscillations with large magnitudes (red and yellow) represent spike-and-wave activities (SW).

**Figure 10:**
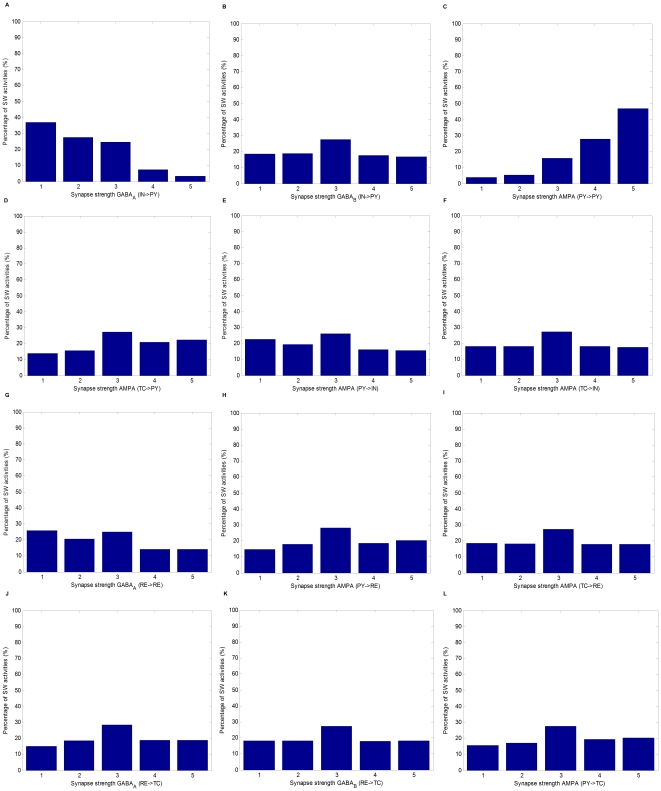
Percentages of SW activities with a given synapse strength for each of the twelve synapses in the network. Y-axis represents the percentages of SW activities. X-axis represents the relative strength of the synapse in terms of five grade levels: 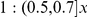, 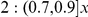, 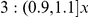, 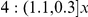, and 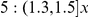, where 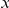 represents the corresponding maximum conductances of receptor channels in the control condition. (A) GABA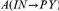. (B) GABA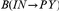. (C) AMPA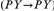. (D) AMPA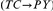. (E) AMPA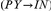. (F) AMPA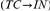. (G) GABA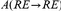. (H) AMPA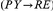. (I) AMPA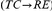. (J) GABA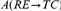. (K) GABA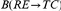. (L) AMPA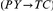.

Among one million simulations, there are a total of 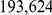 SW activities. The distribution of SW activities in terms of the strength of each of the twelve synapses in the network is shown in Fig. 10. The SW activities cover the full range of tested values of all the synapses and this is also true for spindle activities (not shown). As a result, any synapse with strength in any of the five grade levels could be associated with both pathological and physiological regimes. This further demonstrates the important role played by the combination of parameters in seizure generation. In addition, compared with others, GABA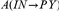 and AMPA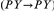 appear to be two critical factors in the range being tested. As shown in Fig. 10A and Fig. 10C, as the strength of GABA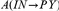 decreases or the strength of AMPA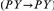 increases, there are more SW activities.

**Figure 11:**
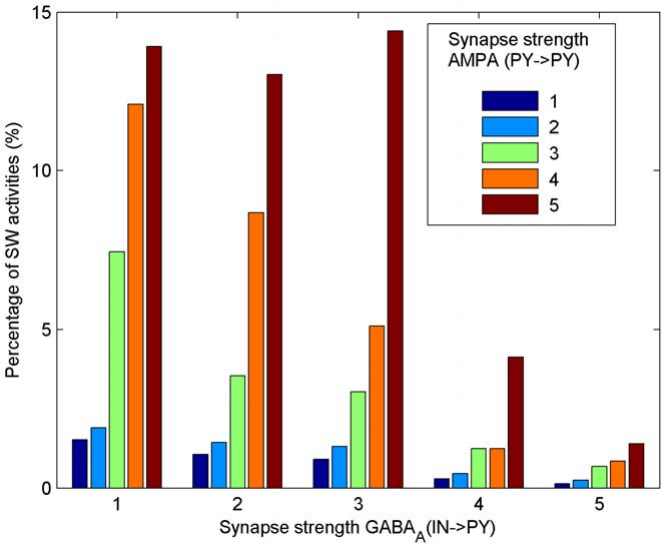
The joint distribution of SW activities in terms of the strength of two critical synapses. X-axis represents the relative strength of GABA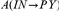. Y-axis represents the percentage of SW activities. Different colors represent the relative strength of AMPA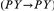 as specified in the legend. The relative strength of both GABA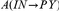 and AMPA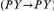 are in terms of five grade levels: 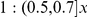, 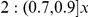, 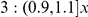, 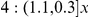, and 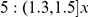, where 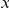 represents the corresponding maximum conductances of receptor channels in the control condition.

Fig. 11 shows the joint distribution of SW activities in terms of the strength of the two critical parameters, GABA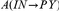 and AMPA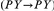. As shown in Fig. 11, although most SW activities occur when GABA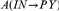 is small and AMPA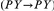 is large, there are a small number of SW activities (represented by the rightmost deep blue bar in Fig. 11), which can even arise with maximal strength of GABA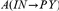 and minimal strength of AMPA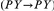. There are a total number of 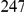 such cases out of 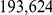 SW activities.

The above results demonstrate that SW activities could arise from a huge number of combinations of underlying parameters and any parameter with values in the full range being tested could be associated with both pathological and physiological regimes. Although some parameters appear to be more critical than others, there is no guarantee that those parameters will always play a leading role in the generation of SW activities. Instead, due to the strong nonlinearity and high complexity of neuron circuits, the underlying mechanisms of SW activities are very complex.

#### Phase flow representations of oscillations

Phase flow analysis has been successfully applied to macroscopic (mean field) models, which are typically described by a few differential equations. However, as the biophysical network model in this paper is described by hundreds of differential equations, it is infeasible to demonstrate a complete phase flow in such high dimensional space. Nevertheless, as neural oscillations are of our interest, two state variables are chosen to obtain phase flow in a two-dimensional subspace: one such state variable is the field potential, an indicator of network-level oscillations, and the other state variable is the intracellular calcium concentration in a reticular cell, an indicator of cellular-level subthreshold oscillations.

**Figure 12:**
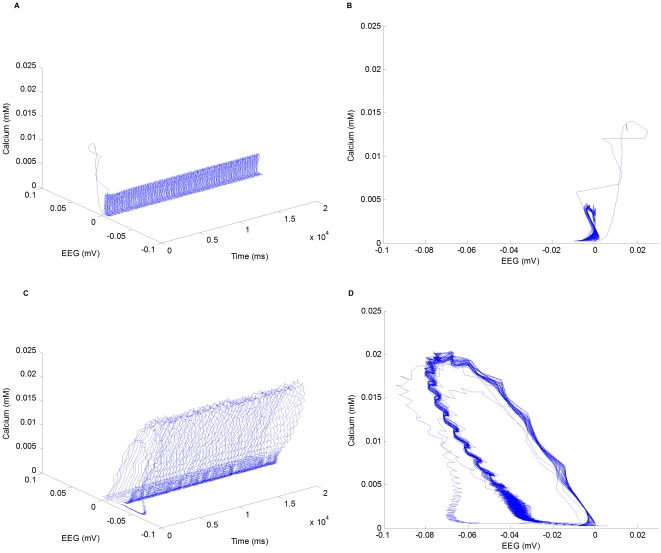
Trajectories and phase flow representations of spindle and SW oscillations in a two-dimensional subspace. (A) Trajectory of spindle oscillation in a 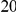-second simulation. (B) Phase flow representation of spindle oscillation (limit cycle). (C) Trajectory of SW oscillation in a 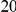-second simulation. (D) Phase flow representation of SW oscillation (limit cycle).

From the resting state, the network may be entrained to oscillation by applying a hyperpolarizing current step to a thalamocortical cell. If the amplitude of the current step is small, the network will display damped oscillation and return to rest in a few cycles. However, if the pulse is large enough, the network will display sustained oscillation. Typically, the sustained oscillation would be either spindle or delta wave depending on the parameter setting of the network. This indicates that there exit a stable equilibrium point corresponding to the resting state and a stable limit cycle corresponding to spindle or spike-and-wave oscillation. Fig. 12 shows the phase flows of spindle and spike-and-wave oscillations in the two-dimensional subspace. The spindle oscillation is obtained from a 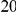-second simulation with the parameters corresponding to the control condition. The spike-and-wave oscillation is obtained with the same set of parameters except that the conductance of GABA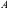 receptors in cerebral cortex is set to be 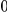. The phase flows in Fig. 12B and Fig. 12D demonstrate the limit cycles corresponding to spindle and SW oscillations, respectively.

**Figure 13:**
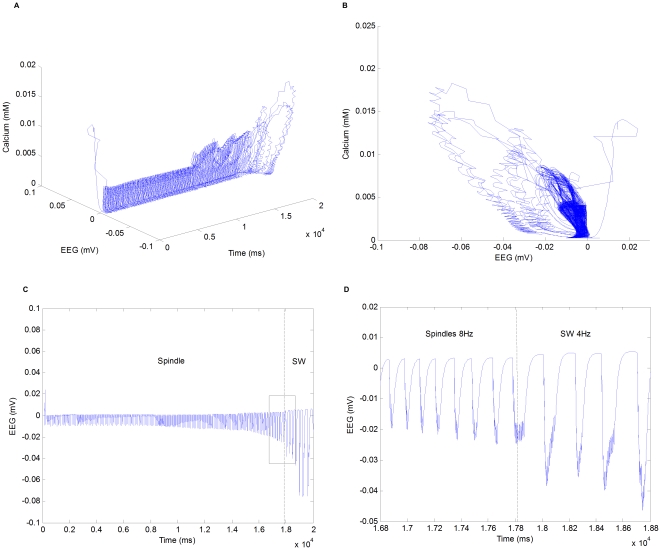
Trajectories, phase flow representations, and field potentials of the transition from spindle to spike-and-wave. (A) Trajectory of the transition in a 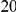-second simulation. (B) Phase flow representation of the transition. (C) Field potentials of the transition in a 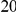-second simulation. (D) A detailed look at the transition by zooming in the field potential.

To demonstrate the transition from spindle to spike-and-wave, we use the conductance of GABA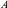 receptors in cerebral cortex, 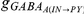, as the control parameter, in a 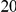-second simulation. At each time step of 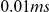, the conductance is decreased by 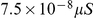. As a result, the control parameter undergoes stepwise linear decay from an initial value of 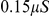 at time 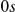 to 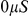 at time 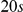. As shown in Fig. 13, the transition from spindle to SW occurs at about 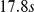. The network is in the mode of spindle oscillation before 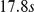 and the amplitude of the limit cycle slightly increases as 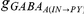 decreases. As shown in Fig. 13D, the oscillation frequency drops from 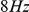 to 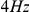 at about 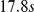 and the network switches into the other mode. In this mode, the oscillation continues at lower frequencies and the amplitude of limit cycle starts to grow dramatically as the control parameter increases. The new mode corresponds to spike-and-wave oscillation. This transition is similar to a supercritical flip (period doubling) bifurcation, which leads to the appearance of a new cycle (SW) with a double period (in the vicinity of the original cycle (spindle) in which the system has lost stability) [53].

### The implication on optimal therapeutic treatments

For a given activity of the network, the underlying parameter combinations form a complex parameter set in a high-dimensional parameter space, where each point in the parameter set of SW oscillations can be considered as a specific *pathological instance*, which corresponds to a particular individual suffering from epilepsy. As a result, the integrative view of mechanisms underlying epileptic seizures has direct implications on the optimal treatments of epilepsies.

To discuss the implication on optimal therapeutic treatments, in this section, we first use simulation to show trajectory activities modified by drug intervention, then introduce the dose-response relationship, define a drug optimization problem, and finally analyze results from the optimization.

#### Trajectory activities modified by drug intervention

In this section, we show trajectory activities modified by drug intervention. We perform 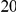-second simulations to demonstrate that the spike-and-wave oscillation (seizure) could be switched back to normal spindle oscillation by modifying different network parameters with time gradually to mimic the effects of different drugs. Initially, the network is set to be oscillating in SW mode with 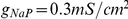 and all the other parameters corresponding to the control condition. When a drug is applied, we assume all the target synapses/ion channels in the network will be proportionally affected.

**Figure 14:**
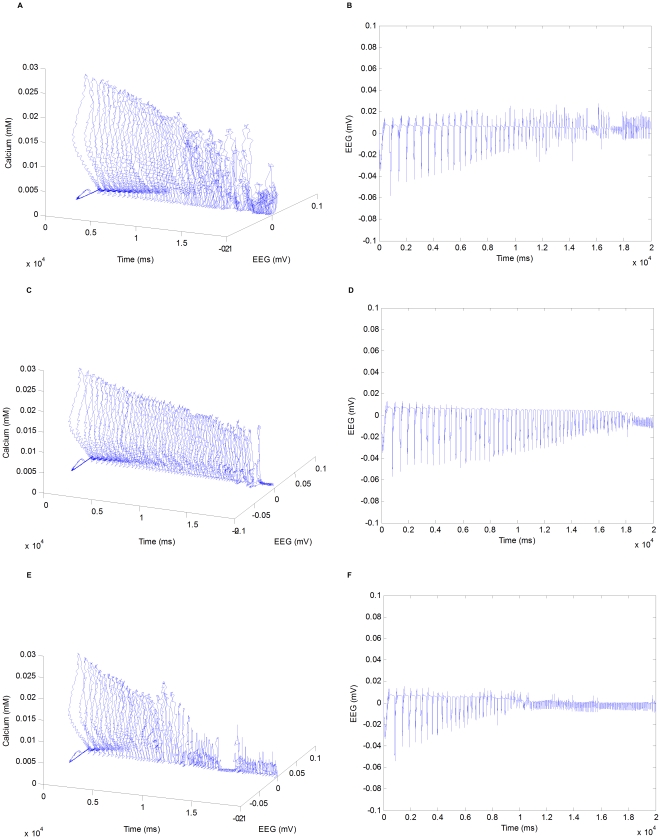
Trajectory activities and field potentials modified by drug intervention in a 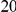-second simulation. (A) Trajectory activities modified by GABA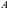 agonists. (B) Field potentials modified by GABA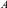 agonists. (C) Trajectory activities modified by AMPA antagonists. (D) Field potentials modified by AMPA antagonists. (E) Trajectory activities modified by GABA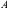 agonists and AMPA antagonists combination. (F) Field potentials modified by GABA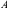 agonists and AMPA antagonists combination.

First, we use the conductances of all the GABA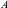 receptor channels as the target parameters to reflect the effect of GABA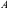 agonists. In the simulation, all the target parameters linearly scale up at each time step from an initial value of 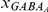 at time 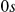 to 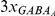 at time 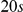, where 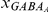 represents the target parameter values in the control condition. As shown in Fig. 14A and Fig. 14B, due to the increase of GABA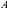 mediated inhibition, the mode of the network is transited from SW back to spindle at about 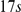.

Second, we demonstrate the effect of AMPA antagonists with all the conductances of AMPA receptor channels as the target parameters. In the simulation, all the target parameters linearly scale down at each time step from an initial value of 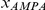 at time 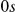 to 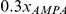 at time 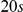, where 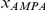 represents the target parameter values in the control condition. As shown in Fig. 14C and Fig. 14D, the mode of the network is transited from SW back to spindle at about 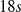 as a result of the decrease of AMPA mediated excitation.

Finally, we show the effect of a combination of GABA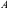 agonists and AMPA antagonists. In this case, the conductances of both GABA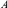 and AMPA receptors are target parameters. While the conductances of all the GABA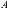 receptor channels scale up linearly from 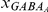 to 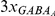 at time 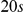, the conductances of all the AMPA receptor channels scale down linearly from 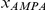 to 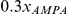, where 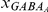 and 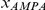 represents the corresponding parameter values in the control condition. As shown in Fig. 14E and Fig. 14F, in this case, the mode of the network is transited from SW back to spindle at about 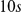, which is much earlier than the previous two cases with each drug alone (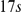 and 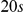, respectively). In fact, the efficacy of the drug degrades significantly as the dose increases. Two low-dose drugs with complementary receptors in combination are likely to produce a greater therapeutic effect a larger dose of a single drug, which will be discussed in the following sections.

#### Dose-response relationship

In general, the concentration of a drug at the site of binding determines its effect. However, the actual dependency can be complex and is often nonlinear. The relationship between the drug dose and the response often empirically follows the shape of a receptor binding curve, which is described by Hill equation [46]

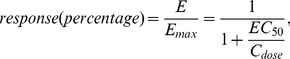
(1)where 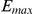 is the efficacy of a drug, which refers to the maximum achievable response, 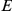 is the actual response achieved at a certain dose level 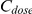, and 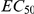 is the half maximal effective concentration of the drug, which induces a response halfway between the baseline and maximum levels after a specified exposure time and is commonly used as a measure of the potency of a drug.

**Figure 15:**
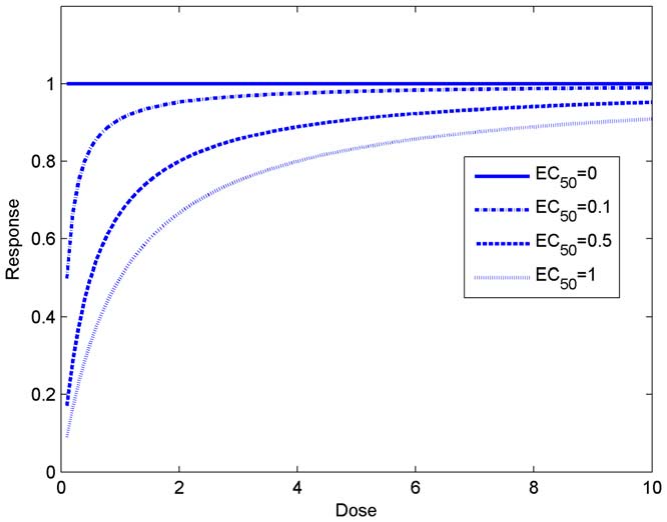
Dose-response relationships with different 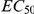 values.

According to the above nonlinear dose-response relationship, if the dose is 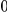, the response will be 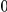; if the dose equals to 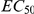, the (percentage) response will be at 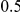; if the dose is 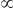, the response will be at 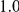. Fig. 15 shows several dose-response curves with different 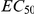 values.

From equation (1) , for a desired response level 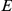, the dose required is given by
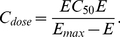
(2)


#### Optimal therapeutic treatments

The toxicity of a drug is dose related. Furthermore, as the tests of toxicity can not be easily performed on humans, dose of the drug is often adopted as a metric for toxicity and it is important to reduce the dose to avoid adverse effects. To quantitatively investigate the optimal therapeutic treatments, we consider an optimization problem as follows.

Given a number of 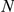 anti-epileptic drugs, the optimal treatment 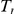 is defined as a combination of the drugs with the minimal total dose such that the epilepsy can be controlled. 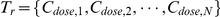 is found by solving the following optimization problem
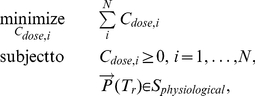
(3)where 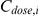 is the dose of the 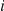-th drug and is an optimization variable, 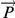 is a vector of parameters characterizing the state of patient in an n-dimensional parameter space, 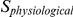 denotes a regime of the parameter space that corresponds to the physiological condition, 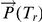 specifies the modified parameter set after applying the treatment.

While the optimization problem (3) is general, as a demonstration, we specifically consider drugs with one of the three complementary mechanisms of action: NaP antagonist, GABA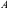 agonist, and AMPA antagonist. When a drug is applied, we assume all the target synapses/ion channels are affected to the same degree. For example, if an AMPA antagonist is applied, the strength of all the AMPA-mediated synapses are proportionally reduced. As a result, the scope of the problem is limited to the three-dimensional subspace depicted in Fig. 8.

Since the working of each drug can be complex, we model the response of a drug by the resulted change of the corresponding scale factor
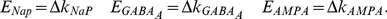
(4)By the same naming convention, as shown in Fig. 8, the sets of points in the physiological and pathological regimes are denoted by 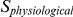 and 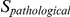, respectively. For a given pathological instance, 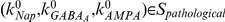, the optimal treatment
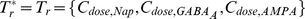
(5)is found by minimizing the total dose
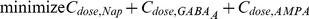
(6)

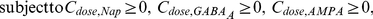
(7)

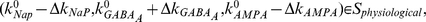
(8)

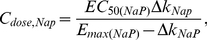
(9)

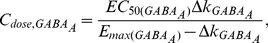
(10)

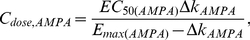
(11)where 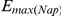, 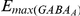, 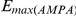, 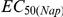, 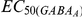, and 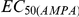 are the efficacies and half maximal effective concentrations of the three drugs, and (9), (10) (11) are based upon (2).

The parameter landscape shown in Fig. 8 provides a basis for solving the drug optimization problem defined above. The optimal drug treatment corresponds to the most “economical” way of moving a pathological instance towards the boundary between the pathologically and physiological regimes. In our experimental study, the E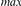 values of all the drugs are set to be a number slightly larger than the corresponding maximum scale factor of the conductance: E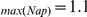, E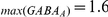, E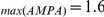, such that the epilepsy of all the patients in the bounded parameter space can be controlled with a finite dose. The default values of 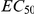 for all the drugs are set to be 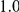. Due to both the limitations of the model and the simplifications made for the drug optimization problem, the numerical optimal solutions are only used to provide important understandings on several key issues, as exemplified below.

#### The effect of drug potencies on the optimal treatments

The potency of each drug is expected to have a significant influence on the choice of the optimal therapeutic treatment. To more clearly see this, we use the persistent sodium NaP antagonist to demonstrate. As the potency of a drug is often measured by its 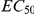, we solve the optimization problem (6) with decreasing values of 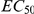: 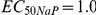, 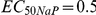, 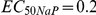, 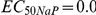.

**Figure 16:**
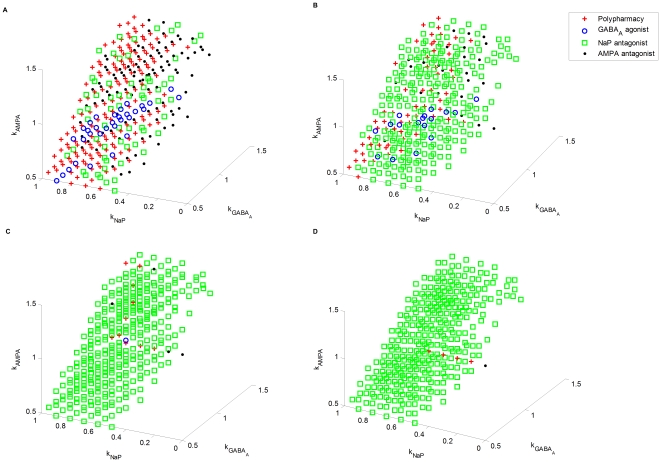
Optimal treatments as functions of the potency of the NaP antagonist 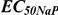. (A) 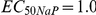. (B) 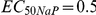. (C) 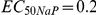. (D) 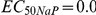.

The resulting optimal drug combinations are shown in Fig. 16. Since there are three candidate drugs, the optimal treatment can be an optimal monopharmacy solution, or a polypharmacy combination involving more than one drug. In the figure, each pathological instance is marked according to its optimal treatment: GABA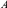 agonist alone (blue circle), NaP antagonist alone (green square), AMPA antagonist alone (black dot), and polypharmacy (red cross).

**Figure 17:**
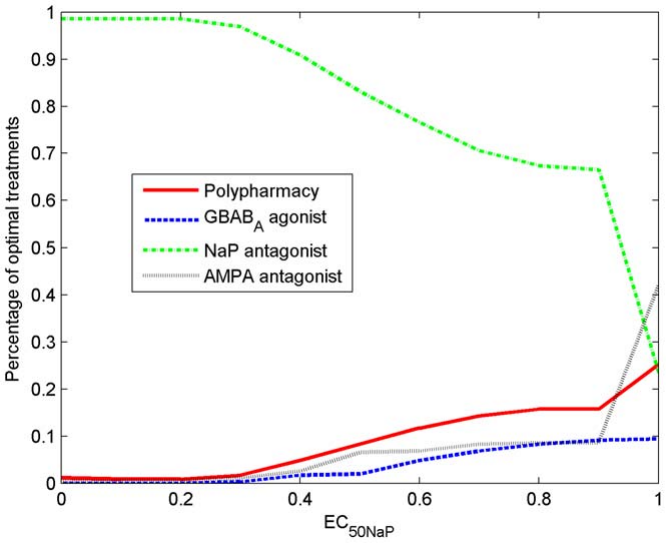
The breakdown of optimal treatments as a function of the potency of the NaP antagonist 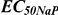.

In Fig. 16, as 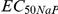 decreases, the potency of the NaP antagonist increases. Accordingly, the number of NaP antagonist monopharmacy based optimal treatment instances increases. The breakdown of optimal treatments as a function of 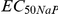 is depicted in Fig. 17. From these results, it is not difficult to see the need for developing high-potency drugs.

In addition to the issue of drug efficiency discussed above, it is also worthy noting a related aspect, *treatability*. This is the best illustrated by the results where 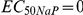. In this case, the potency of the NaP antagonist is effectively infinite. However, the set of optimal treatments does not exclusively consists of the NaP antagonist and there is a case of AMPA antagonist as in Fig. 16D. A detailed examination reveals that the pathological set contains instances that are located on the plane defined by 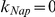. For these instances, it is evident that the existence of persistent sodium current is not the root cause for the occurrence of seizure, which is in fact exclusively generated by the other two factors 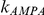 and 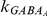. As such, the epilepsy of patients represented by those points cannot be controlled by suppressing 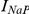 alone. This again underscores the importance of correct identification of the underlying mechanisms that are responsible for seizure generation and personalized treatment.

#### The effect of nonlinear dose-response characteristics and the efficacy of polypharmacy

**Figure 18:**
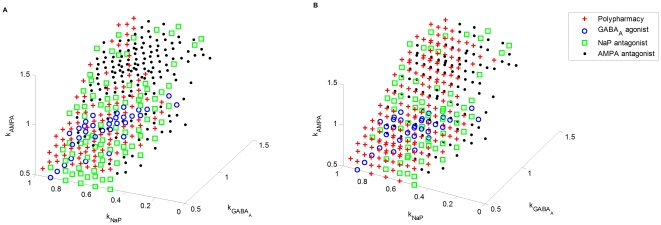
The effects of linearity and nonlinearity of dose-response relationships. (A) Linear dose-response relationship. (B) Nonlinear dose-response relationship.

The nonlinearity of the dose-response relationship also has a large impact on the choice of the optimal treatments. Many dose-response curves follow the shape of a receptor binding curve, where the efficacy of the drug degrades significantly as the dose increases. For instance, the dose required to achieve 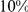 of a response may be much less than one ninth of that required to achieve 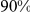 of the response. Such non-linearity implies that two low-dose drugs with complementary receptors in combination are likely to produce a greater therapeutic effect with less risk of toxicity than a larger dose of a single drug. To demonstrate this, we solve the same optimization problem with a linear dose-response relationship, i.e.,
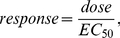
(12)and the results are given in Fig. 18A. In this case, the number of optimal treatments based on polypharmacy is significantly decreased as compared with the nonlinear case shown in Fig. 18B.

This partially explains why combining drugs with different mechanisms of action may be more beneficial in efficacy and toxicity than a combination of drugs with similar mechanisms of action, such as two sodium channel drugs [54]. Our optimization results are in line with the clinical observation that polypharmacy with drugs acting on complementary receptor sites may produce good potentiating effect. Note that, in the analysis above, each drug is assumed to have only one site of action. However, in practice, even one drug can have multiple sites of action. Therefore, the optimization results above are also in line with the clinical observation that the most efficient drugs are the drugs with multiple sites of action.

More broadly, there has been widespread debate about the effect of polypharmacy for the treatment of epilepsy. As hinted earlier, some people favor the use of multiple drugs with a lower dose for each and believe this is more beneficial than administrating a single high-dose drug. Our foregoing discussion offers one possible explanation for this by attributing it to the nonlinear dose-response characteristics of typical drugs. Other practitioners believe that a full dose of one drug achieves better control of seizures with fewer adverse effects. The results in Fig. 16 possibly provides a more complete picture: while monopharmacy is often sufficient (may not be optimal) to control the epilepsy of patents represented by points close to the boundary of the pathological regime, polypharmacy is often more efficient for patients with intractable epilepsy, which are represented by points deeply inside the pathological regime. Note that our results are obtained based upon simplifying assumptions. Polypharmacy is a clinically complex matter. Pharmacodynamic or pharmacokinetic drug-drug interactions may exist. To simplify the matter, we have assumed that the effects of multiple drugs are addictive. More complete analysis with consideration of drug-drug interactions will be a subject of the future work when additional modeling and experimental data is available.

Nevertheless, with the current model setup, the presented parameter space landscape and optimization results provide additional insights on the issue of polypharmacy. In addition to the nonlinear dose-response relationships, the location of each pathological instance, relative to the boundary of the pathological and physiological regimes, plays an important role in judging the benefit of polypharmacy. Furthermore, the distribution of pathological instances provides a “view” of the entire patient population. Since the distribution of patients is not uniform and more patients are located close to the boundary, one drug may be adequate for the majority of patients. On the other hand, rational polypharmacy may be very beneficial for a small population of patients. These observations are in agreement with typical clinical trials.

#### The variability of optimal treatments and the needs for personalized therapy

From the above study, it is clearly seen that the optimal therapeutic treatment varies greatly among patients. Depending on the individual state, optimal monopharmacy or polypharmacy may be the most desirable. Variability is not only in terms of the choice of drugs but also in terms of doses. Low dose may be sufficient for patients whose model parameters are close to the boundary of the pathological and physiological regimes while high dose may be needed for others. It is also possible that, due to toxicity limitation, the epileptic activities of some patients represented by points deeply inside the pathological regime cannot even be controlled by pharmaceutical means. In this case, epilepsy surgery becomes necessary. In addition, although polypharmacy may be very effective for patients deeply inside the pathological regime, the best drug combination and the dose for each selected drug must be carefully chosen. While many road blockers still exist, there is clearly a need towards personalized therapy. To achieve this goal in the future, computer models and simulation may be leveraged to provide computational aids to help clinicians determine the optimal treatments.

## Materials and Methods

All the results in this paper are obtained with Matlab 7.1 and the simulation can be downloaded from ModelDB(senselab.med.yale.edu/modeldb). As shown in Fig. 1, the thalamocortical network model in this study is similar to the one adopted in [5], [7]. The network includes the single compartment representations of four types of cells: cortical pyramidal (PY) cells, cortical interneurons (IN), thalamic reticular (RE) cells, and thalamocortical (TC) cells. The network model includes eight cells with two cells for each type. Albeit its simplicity, the network model has been proven to be able to make predictions for larger networks with similar structures [7]. The single-compartment models include intrinsic and synaptic currents described by the following equation
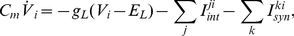
(13)where 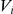 is the membrane potential, 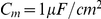 is the specific capacity of the membrane, 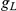 is the leakage conductance, 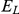 is the leakage reversal potential, and intrinsic and synaptic currents are represented by 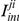 and 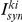, respectively.

Intrinsic currents 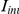 are modeled by kinetic models of the Hodgkin and Huxley type [55] described by the following equations
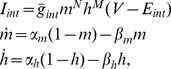
(14)where 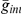 is the maximal conductance, and 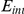 is the reversal potential. The gating properties of the current are dependent on 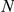 activation gates and 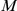 inactivation gates, with 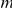 and 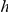 representing the fraction of gates in open form and with respective rate constants 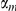, 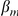, 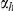, and 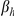. Rate constants are dependent on either membrane voltage or intracellular calcium concentration.


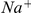
 and 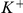 currents contribute to action potentials and are included in all the cell models [56]. IN cells produce “fast-spiking” [57] firing and contain no other current than is necessary for action potentials. For the pyramidal cells, one additional slow voltage-dependent 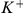 current (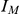) [58] producing “regular-spiking” pattern characterized by adaptation is modeled [57]. There are T-currents in the model of thalamic cells such that bursts of action potentials can be produced. The T-current in RE cells 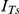 are of slow kinetics, which is given in [59], [60]. The T-current in TC cells 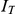 is modeled by kinetics similar to the model of [59] with activation considered at steady state and inactivation described by a first order equation [5]. In addition to 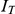, TC cells also include leak potassium current 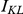 and hyperpolarization-activated inward current 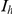 [5]. We only consider the voltage dependence of 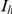 and do not include the upregulation of 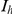 by intracellular Ca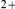 which leads to wax-and-wanning properties. Intracellular 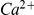 dynamics are modeled by a simple first-order decay with a time constant of 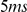. The equilibrium 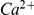 concentrations are 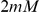 extracellularly and 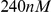 intracellularly, which corresponds to a reversal potential of 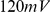 [5], [61].

The cell membrane areas of all cell types and the conductance values of the corresponding intrinsic currents are as follows: for cortical pyramidal cells, 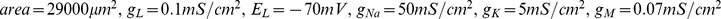; for cortical interneurons, 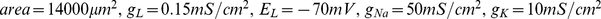; for thalamic reticularcells,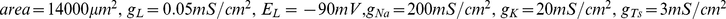; for thalamocortical cells, 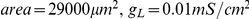, 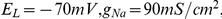 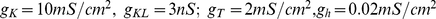. In addition, we also add persistent current 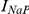, high threshold calcium 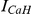, and afterhyperpolarization current 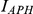 to cortical pyramidal cells to study the roles of intrinsic currents in the generation of seizures. The corresponding conductances are treated as free parameters in the exploration of parameter space.

The synaptic current 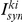 from presynaptic cell 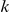 to postsynaptic cell 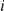 is simulated by activating a pulse of transmitter when cell 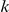 fires an action potential. As shown in Fig. 1, the receptor types present in synaptic connections are as follows: all excitatory connections (TC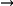 RE, TC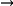 IN, TC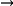 PY, PY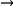 PY, PY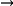 IN, PY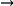 RE, PY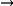 TC) are mediated by AMPA receptors; some inhibitory connections 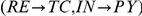 are mediated by a mixture of GABA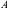 and GABA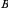 receptors, whereas intra-RE connections are mediated by GABA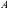 receptors. In the control condition, the receptor conductance values displaying spindle oscillations are 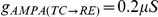, 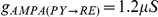, 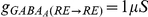, 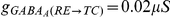, 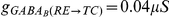, 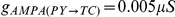, 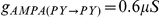, 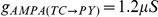, 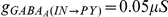, 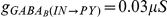, 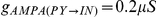, 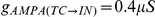.

The models of AMPA and GABA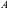 receptors are described by the following equation [62]

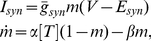
(15)where 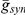 is the maximal conductance, 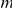 is the fraction of the open receptors, 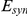 is the reversal potential, 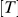 is the transmitter concentration in the cleft, and 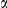 and 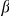 are forward and backward binding rate constants of 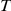 to open the receptors. The parameters used are as follows: 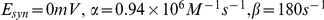 for AMPA receptors and 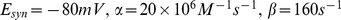 for GABA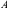 receptors.

Due to the nonlinear property, the models of GABA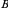 receptors are described by different equations [63]

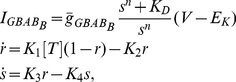
(16)where 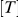 is the GABA concentration in the synaptic cleft, 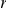 is the fraction of GABA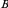 receptors in the activated form, 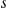 is the normalized G-protein concentration in activated form, 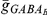 is the maximal postsynaptic conductance of 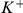 channels, 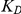 is the dissociation constant of G-protein binding on 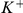 channels, 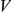 is the postsynaptic membrane potential, and 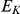 is the equilibrium potential for 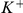. The parameters are as follows: 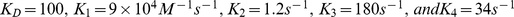, with 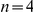 binding sites.

The field potentials (EEG) are simulated based on postsynaptic and intrinsic currents of the two pyramidal cells
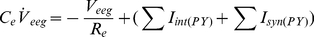
(17)where 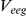 represents the field potentials (EEG waveforms), 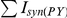 represents the sum of postsynaptic currents of the two PY cells, 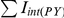 represents the sum of intrinsic currents of the two PY cells, 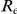 and 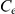 represent the resistance and capacitance of extracellular media and EEG equipment, which behave like low-pass filters. Note that, the intrinsic currents do not include the 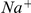 and 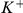 currents for action potentials generation as they make little contribution to the EEG [64].

Generally, if there are N pyramidal cells in the model, the field potential should be computed based on N pyramidal cells (a weighed sum of currents of the PY cells and the weight is determined by the distance between each PY cell and the position where the field potential is measured). In this paper, there are only two PY cells in the eight cell model. The sum of currents from the two PY cells corresponds to the field potential measured in a position with equal distance from each PY cell.
